# Molecular Mechanism Based on Histopathology, Antioxidant System and Transcriptomic Profiles in Heat Stress Response in the Gills of Japanese Flounder

**DOI:** 10.3390/ijms23063286

**Published:** 2022-03-18

**Authors:** Weijie Yan, Yingjie Qiao, Jiayi He, Jiangbo Qu, Yuxiang Liu, Quanqi Zhang, Xubo Wang

**Affiliations:** Key Laboratory of Marine Genetics and Breeding, Ministry of Education, Ocean University of China, Qingdao 266005, China; sc141ywj@163.com (W.Y.); 17854173828@163.com (Y.Q.); 13210236844@163.com (J.H.); qujiangbo@stu.ouc.edu.cn (J.Q.); liuyuxiang@stu.ouc.edu.cn (Y.L.); qzhang@ouc.edu.cn (Q.Z.)

**Keywords:** Japanese flounder, heat stress, oxidative stress, gill

## Abstract

As an economically important flatfish in Asia, Japanese flounder is threatened by continuously rising temperatures due to global warming. To understand the molecular responses of this species to temperature stress, adult Japanese flounder individuals were treated with two kinds of heat stress—a gradual temperature rise (GTR) and an abrupt temperature rise (ATR)—in aquaria under experimental conditions. Changes in histopathology, programmed cell death levels and the oxidative stress status of gills were investigated. Histopathology showed that the damage caused by ATR stress was more serious. TUNEL signals confirmed this result, showing more programmed cell death in the ATR group. In addition, reactive oxygen species (ROS) levels and the 8-O-hDG contents of both the GTR and ATR groups increased significantly, and the total superoxide dismutase (T-SOD) activities and total antioxidant capacity (T-AOC) levels decreased in the two stressed groups, which showed damage to antioxidant systems. Meanwhile, RNA-seq was utilized to illustrate the molecular mechanisms underyling gill damage. Compared to the control group of 18 °C, 507 differentially expressed genes (DEGs) were screened in the GTR group; 341 were up-regulated and 166 were down-regulated, and pathway enrichment analysis indicated that they were involved in regulation and adaptation, including chaperone and folding catalyst pathways, the mitogen-activated protein kinase signaling (MAPK) pathway and DNA replication protein pathways. After ATR stress, 1070 DEGs were identified, 627 were up-regulated and 423 were down-regulated, and most DEGs were involved in chaperone and folding catalyst and DNA-related pathways, such as DNA replication proteins and nucleotide excision repair. The annotation of DEGs showed the great importance of heat shock proteins (HSPs) in protecting Japanese flounder from heat stress injury; 12 *hsp* genes were found after GTR, while 5 *hsp* genes were found after ATR. In summary, our study records gill dysfunction after heat stress, with different response patterns observed in the two experimental designs; chaperones were activated to defend heat stress after GTR, while replication was almost abandoned due to the severe damage consequent on ATR stress.

## 1. Introduction

Environmental stress has an influence on many biological functions and disturbs homeostasis [1]. Temperature is one of the most important environmental factors which can affect the food intake, growth, maturation, reproduction, behavior, physiology, metabolism and even survival of economic teleosts [2,3,4,5,6,7,8,9,10,11]. Appropriate temperatures may promote the growth of fish, while temperatures above the optimum boundary will have negative impacts [12]. Fish have contrapuntally developed several levels of biochemical responses, including primary (initial neuroendocrine responses), secondary (cellular reactions, immune function and metabolism) as well as tertiary responses (growth, feeding and other whole body performance changes) [13,14]. Up to now, the average seawater temperature has increased by 0.6 °C from the late 19th century and is expected to increase by 2–4 °C further in the early 22nd century [15,16]. As a result of the severe global warming situation, a great number of studies have focused on the impact of water temperature on both freshwater and marine fish [17,18,19,20].

Heat stress could influence the metabolic rate, destroy heme groups and elevate the production of reactive oxygen species (ROS) which play a critical role in some important cellular events, including signaling, apoptosis and cell growth [21,22,23]. Moderate concentrations of ROS are required during normal physiological activity; approximately 0.1–0.2% of the oxygen consumed by aerobic cells is transformed to ROS, which is essential for maintaining the regular function of cells, including resisting pathogens [24,25]. However, the excess production of reactive oxygen species and the imbalance of antioxidant system activity may lead to oxidative stress and DNA, lipid and protein damage, resulting in a series of cellular dysfunctions [26,27,28,29]. Furthermore, oxidative stress could cause cellular apoptosis, a kind of programmed cell death which plays an important role in removing dying or infected cells [30,31,32]. To eliminate ROS and counteract the adverse effect of oxidative stress, cells have developed an antioxidant defense system, including low-molecular weight scavengers and enzyme defenses [33]. Superoxide dismutase (SOD), catalase (CAT), peroxidase (POD), glutathione peroxidase (GSH-Px) and glutathione reductase (GR) are major antioxidant enzymes that maintain the normal redox state of the cell [34].

In addition to the antioxidant defense system, heat shock proteins (HSPs) respond actively to heat stress [35]. HSPs were first reported in the heat shock response of drosophila and were found in the heat shock response of a great number of aquatic animals, including *Siniperca chuatsi*, *Oncorhynchus mykiss*, *Litopenaeus vannamei*, *Pomacea canaliculata*, *Ciona savignyi*, *Danio rerio* and *Paralichthys olivaceus* [36,37,38,39,40,41,42,43]. Additionally, mitogen-activated protein kinase (MAPK) pathways were proved to respond actively to extracellular environmental signals, including heat stress [44,45,46]. In addition, previous studies also demonstrated that the induction of *hsp* genes was associated with *mapk* gene activation under conditions of stress in aquatic animals [47,48]. In teleosts, the identification of *mapk* genes and expression analysis in embryos and unchallenged individuals have been reported decades ago [49,50]. In a previous study, *p38 mapk* genes were proved to participate in the heat stress response in red blood cells of gilthead sea bream [51]. However, systematic analyses of the roles of important antioxidant genes in the heat shock reactions of Japanese flounder (*Paralichthys olivaceus*) are still lacking.

Japanese flounder is a famous economic fish broadly cultivated in Asia, including the Bohai Sea, the South China Sea, the Yellow Sea, North Korea and the east of Japan [52,53]. As a kind of warm water fish with a temperature range of 15–25 °C, Japanese flounder could hardly survive above 28 °C [54,55]. Nowadays, rising marine temperatures have influenced the survival, growth, distribution, and reproduction of this species [56,57,58]. Numerous studies have illustrated the effect of heat or cold stress on the masculinization, sex-manipulation, energy metabolism, neurosecretion, stress-related gene expression and AMPK regulation mechanism of Japanese flounder [54,55,57,59,60]. As a critical functional organ in fish, the gill is involved in a number of physiological processes, including respiration, waste nitrogen excretion, iono-/osmoregulation, immune response and acid–base balance [61,62]. Due to continuous exposure to the ambient environment, gills are direct targets of stress responses and are more sensitive than other organs to high temperatures [63,64]. Additionally, transcriptome dynamics have been applied frequently in the studies of thermoregulation of Japanese flounder [65,66,67]. Previous research has reported that heat stress could cause histological damage to gills and enzymatic responses, but these studies were separate and focused on single points of damage [68]. To date, integrated observation of the observed damage to molecular mechanisms has not yet been reported. In this study, Japanese flounder were subjected to two kinds of heat stress (a gradual temperature rise and an abrupt temperature rise) to observe the histological damage to gill tissue and antioxidant system enzyme activities, as well as apoptosis status. We hypothesize that heat stress could cause damage to the gill tissue, influence the antioxidant system, and increase the apoptosis level. To further illustrate the molecular mechanisms of oxidant damage, transcriptome data were analyzed. These results will provide a fundamental reference for oxidant damage caused by heat stress in gill tissue. In addition, we also observed different response patterns under the two kinds of heat stress and put forward two hypotheses (slight damage and active repair efforts after GTR, as well as severe damage and near abandonment of repair after ATR) to explain the differences, which will help to further understanding of heat-resistance mechanisms in Japanese flounder.

## 2. Methods

### 2.1. Ethics Statement

This study was conducted in accordance with the Institutional Animal Care and Use Committee of the Ocean University of China and the China Government Principles for the Utilization and Care of Vertebrate Animals Used in Testing, Research and Training (State Science and Technology Commission of the People’s Republic of China for No. 2, 31 October 1988; http://www.gov.cn/gongbao/content/2011/content_1860757.htm, revised in 8 November 2011).

### 2.2. Experiment Design and Sampling

Although individuals from the wild are more suited to this kind of experiment, owing to the difficulties in obtaining wild individuals, fish from a market were utilized instead. A total of 27 Japanese flounder individuals (average body weight 866 ± 166 g, average total length 33 ± 4.5 cm) were collected from Nanshan market, Qingdao, China, and acclimatized under laboratory conditions (seawater at 18 °C, a photoperiod of 14:10 h light:dark, 30 ppt salinity) for one week. The seawater for breeding was refreshed periodically, two times daily. Then, the experimental individuals were randomly distributed into three groups: (1) in the control (C) group, the temperature was held at 18 °C until the end of the experiment; (2) the water temperature of the Gradual Temperature Rise (GTR) group was elevated from 18 °C to 29 ± 1 °C at 1 °C per hour and sampling was performed after 12 h’s stress; (3) the individuals in the Abrupt Temperature Rise (ATR) group were transferred to 28 °C abruptly and conditions were maintained for 8 h. There were 9 individuals in each group. Professional aquatic glass heaters (SUNSUN, Zhoushan, China, JRB230, 300 W, aquired in Qingdao, China) were utilized to control the temperature. At the end of the heat shock experiment, individuals were euthanized by MS-222 and 3 individuals from each aquarium were randomly sampled for further study.

### 2.3. RNA Isolation, Library Preparation and Sequencing

Following standard protocol, gill samples were immediately frozen in liquid nitrogen after sampling, then total RNA was extracted using TRIzol Reagent (Invitrogen, Waltham, MA, USA), and RNase-free DNase I (TaKaRa, Beijing, China) was applied to remove genomic DNA contamination. The quality and quantity of total RNA were detected by 1.5% agarose gel electrophoresis and spectrophotometry, respectively, and then used for library preparation and sequencing. The transcriptome sequencing was carried out on an Illumina Hiseq 4000 platform of the Beijing Novogene company.

### 2.4. Transcriptome Annotation and Analysis

After trimming adaptors and removing low-quality reads, the clean reads were screened against the complete genome of Japanese flounder (Zhang, unpublished data). The transcripts per kilobase million (TPM) were used to estimate gene expression levels in this study. Analyses of differentially expressed genes (DEGs) between the control group and the two heat-stressed groups were carried out using DEseq2; the threshold for DEGs was *q*-value < 0.05 and |log_2_Foldchange| > 1. Then, the R package (clusterProfiler 4.0) were utilized for enrichment analysis, including Gene Ontology (GO), Kyoto Encyclopedia of Genes and Genomes (KEGG) and Gene Set Enrichment Analysis (GSEA) [69,70,71]. In our previous studies, genome-wide identification of *hsp10/20/40/60* family members and their systematic bioinformatic analyses were performed; the expression profiles of the four subfamilies above and the *hsp70* subfamily were analyzed according to our transcriptome data [72,73,74].

### 2.5. Histological Analysis

Bouin’s fluid was utilized for the preservation of gill samples and dehydrated by a conventional alcohol gradient of 80%, 90%, 95% and 100% alcohol. After transparentizing with xylene and alcohol mix, the samples were embedded in paraffin, sliced with thicknesses of 5 μm and then dewaxing and H&E stain were performed in the traditional way. The samples sealed with neutral gum were finally photographed using a microscope.

### 2.6. Determination of Antioxidant System Enzyme Activities

The gill samples were homogenized with 0.9% saline (1 g gill tissue: 0.9 mL saline) using an electric homogenizer, then the mix was centrifugated with a refrigerated centrifuge (4 °C, 12,000 r/min) and the supernatant was utilized for the antioxidant system enzyme activities analysis. ROS and 8-O-hDG content, T-SOD activity, and total antioxidant capacity (T-AOC) level were determined using visible light reagent kits according to the standard protocols (Nanjing Jiancheng Sci-Tech Co., Ltd., Nanjing, China).

### 2.7. Apoptosis Analysis

The gill tissues from three groups were subjected to terminal deoxynucleotidyl transferase deoxy-UTP nick end labeling (TUNEL) assay (One-Step TUNEL Apoptosis Assay Kit, Beyotime Biotechnology, Shanghai, China) to detect apoptosis. Briefly, dewaxed and rehydrated tissue sections were treated with proteinase K for 25 min at 37 °C, rinsed twice with phosphate-buffered saline (PBS) and then stained with TUNEL reaction mixture for 60 min, followed by counterstaining with DAPI (4′,6-diamidino-2-phenylindole). Finally, the tissue sections were observed under a fluorescence microscope.

### 2.8. Statistical Analysis

All data were presented as the means of each group. We analyzed differences between the two heat-stressed groups and the control group utilizing one-way analysis of variance (ANOVA) in SPSS 20.0; *p* < 0.05 was considered significant. Then, GraphPad Prism 7 was used to visualize the results.

## 3. Results

### 3.1. Effects of Heat Stress on Gill Damage

**Figure 1:**
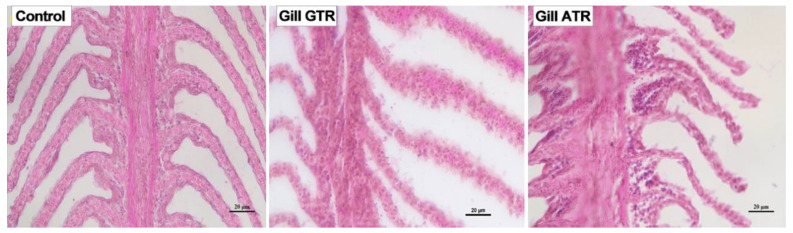
Effect of heat stress on gills of Japanese flounder. GTR, Gradual Temperature Rise; ATR, Abrupt Temperature Rise.

An intact tissue structure and no damage could be observed in the C group (Figure 1, Control), while the GTR group showed swelling of gill lamellae and epithelial cells which hinted at slight damage (Figure 1, Gill GTR). In addition, deformation, serious swelling of the gill lamellae and obvious fusion of epithelial cells were found in the ATR group (Figure 1, Gill ATR). In brief, both kinds of heat stress led to gill damage, especially in the ATR group.

### 3.2. Antioxidant System Enzyme Activities

**Figure 2:**
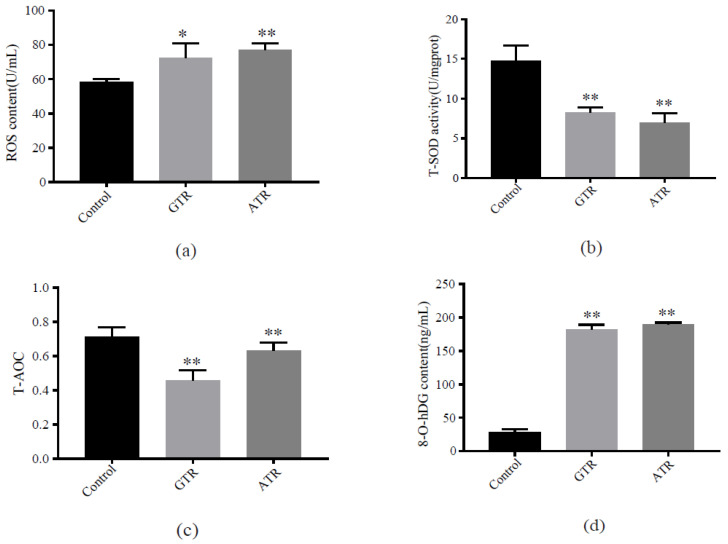
Effect of heat stress on antioxidant system and DNA damage product in the gills of Japanese flounder under two kinds of heat stress. Subfigure (**a**–**d**) represents ROS content, T-SOD activity, the level of T-AOC and 8-O-hDG content, respectively. One asterisk represents significant differences (*p* < 0.05) while two asterisks represent extremely significant differences (*p* < 0.01) between the control group and an experimental group.

The contents of ROS increased (*p* < 0.05) after GTR stress and significantly increased (*p* < 0.01) after ATR stress (Figure 2a). T-SOD activities (Figure 2b) and T-AOC levels (Figure 2c) decreased significantly (*p* < 0.01) after both kinds of heat stress. The 8-O-hDG contents increased significantly (*p* < 0.01) after both ATR and GTR (Figure 2d).

### 3.3. Programmed Cell Death Levels

**Figure 3:**
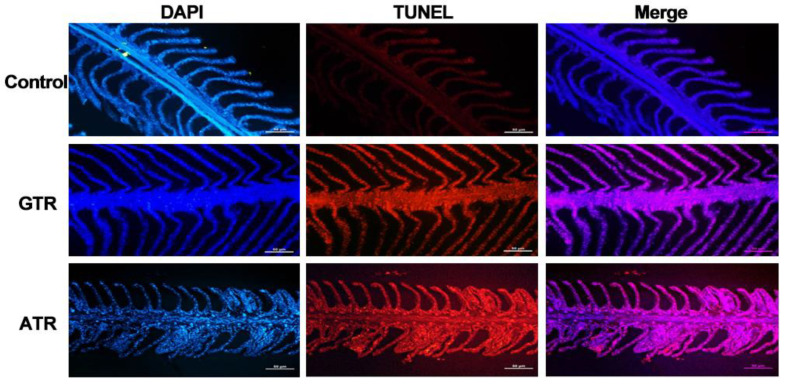
Programmed cell death evaluated by TUNEL (terminal deoxynucleotidyl transferase deoxy-UTP nick end labeling) and DAPI (4′,6-diamidino-2-phenylindole) after heat stress. C, Control; GTR, Gradual Temperature Rise; ATR, Abrupt Temperature Rise.

To evaluate the programmed cell death levels associated with the two kinds of heat stress, TUNEL (red signal) and DAPI (blue signal) were utilized to label DNA fragments and normal cell nuclei, respectively. As shown in Figure 3, there was almost no blue signal in the control group, while there were more blue signals in the GTR and ATR groups, and the signal in the ATR group was the strongest. This result indicated that heat stress caused programmed cell death in the gills of Japanese flounder and that severe stress led to more cell death.

### 3.4. Transcriptional Analysis after Heat Stress

#### 3.4.1. Summary of Preprocessing

A summary of transcriptome sequencing data preprocessing is presented in Supplementary Table S1; every library has over 6 Gb counts, which were all deposited with the National Center for Biotechnology Information (NCBI) with the accession numbers PRJNA716811, PRJNA717095 and PRJNA776544. The Q20 base percentage was above 97.37% and the Q30 base percentage was above 92.99%. Additionally, all the clean reads were mapped successfully to the Japanese flounder genome, indicating that the clean data was of high quality and fit for further analysis.

#### 3.4.2. Gene Expression Patterns and Differential Gene Expression Analysis

**Figure 4:**
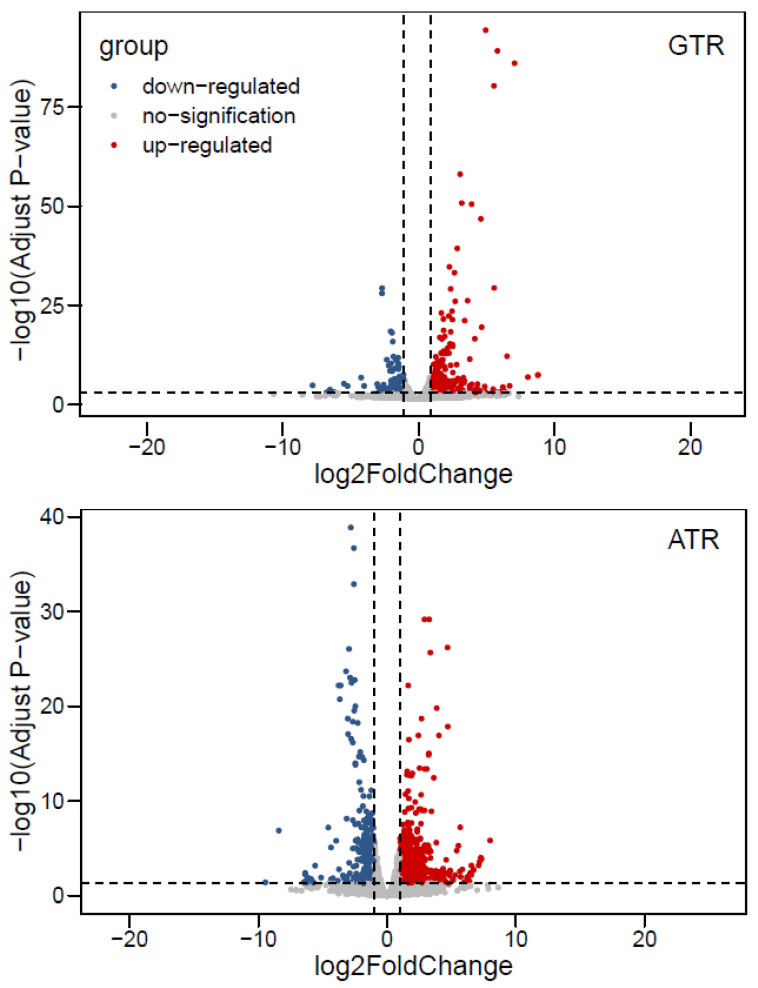
Volcano plot of DEGs after GTR and ATR. Every dot represents one gene; the red dots represent significantly up-regulated genes, the blue dots represent significantly down-regulated genes and the grey dots represent genes which have not reached the differential expression level.

After setting the threshold value as *q*-value < 0.05 and |log_2_Foldchange| > 1, a total of 1567 DEGs were screened after the two kinds of heat stress. As listed in Table 1, 341 genes were found to be up-regulated and 166 genes were found to be down-regulated in the GTR group and the C group, while 627 genes were found to be up-regulated and 423 genes were found to be down-regulated in the ATR group and the C group. The volcano plots of DEGs after GTR and ATR are shown in Figure 4.

#### 3.4.3. GO Enrichment and KEGG Pathway Analysis

As for the GO analysis, after GTR stress, 9 ‘biological process’ categories were enriched, while 13 categories were enriched after ATR stress, including 4 ‘biological process’, 3 ‘molecular function’ and 6 ‘cellular component’ categories. In summary, the same GO terms of ATR and GTR stress were DNA-related, and the GTR stress enriched up-regulated protein refolding terms while the ATR group enriched up-regulated swimming behavior. Detailed information is shown in Table 2 and Table 3.

As for the KEGG enrichment, after GTR stress, 9 pathways were enriched, including 3 up-regulated pathways and 6 down-regulated pathways. As for the ATR stress, 10 pathways were significantly enriched, including 3 up-regulated pathways and 7 down-regulated pathways. Detailed information about all the KEGG pathways is shown in Table 4 and Table 5.

#### 3.4.4. GSEA Results

**Figure 5:**
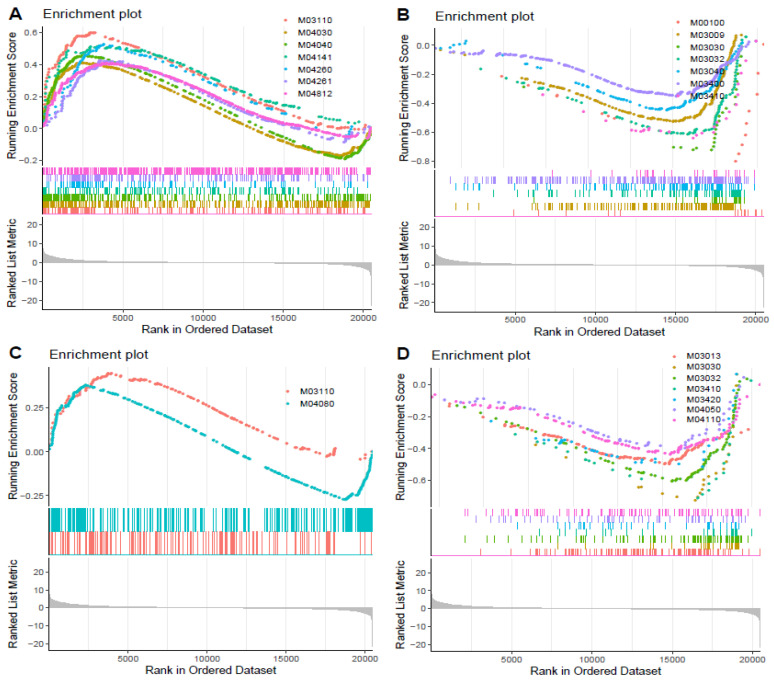
The visualized results of GSEA. The up-regulated pathways after GTR (**A**) are as follows: Chaperones and folding catalysts (M03110), G protein-coupled receptors (M04030), Ion channels (M04040), Protein processing in endoplasmic reticulum (M04141), Cardiac muscle contraction (M04260), Adrenergic signaling in cardiomyocytes (M04261), Cytoskeleton proteins (M04812). The down-regulated pathways after GTR (**B**) are as follows: Steroid biosynthesis (M00100), Ribosome biogenesis (M03009), DNA replication (M03030), DNA replication proteins (M03032), Spliceosome (M03040), DNA repair and recombination proteins (M03400), Base excision repair (M03410). The up-regulated pathways after ATR (**C**) are as follows: Chaperones and folding catalysts (M03110), Neuroactive ligand–receptor interaction (M04080). The down-regulated pathways after ATR (**D**) are as follows: RNA transport (M03013), DNA replication (M03030), DNA replication proteins (M03032), Base excision repair (M03410), Nucleotide excision repair (M03420), Cytokine receptors (M04050), Cell cycle (M04110).

**Figure 6:**
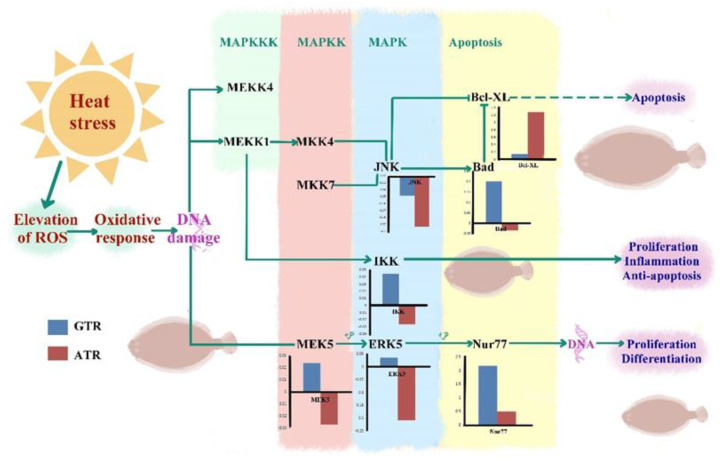
The response of the mitogen-activated protein kinase signaling (MAPK) pathway after two kinds of heat stress. Note: The blue bar represents the GTR while the red bar represents ATR; the up-regulated genes are above the line while the down-regulated genes are below the line.

As for GTR stress, 16 KEGG pathways were significantly enriched (*p* < 0.05, adjust *p* < 25%) in the GTR vs. C group, including 7 up-regulated gene sets as well as 9 down-regulated gene sets. We selected seven gene sets to conduct a visualized analysis, including two up-regulated gene sets, namely, the MAPK signaling pathway (KEGGID:M04040) and chaperones and folding catalysts (KEGGID:M03110) (Figure 5A), as well as 5 down-regulated gene sets, namely, DNA replication proteins (KEGGID:M03032), spliceosome (KEGGID:M03040), DNA replication (KEGGID:M03030), base excision repair (KEGGID:M03410) and DNA repair and recombination proteins (KEGGID:M03400) (Figure 5B). Detailed information is shown in Supplementary Table S2. In addition, the visualized expression patterns of the MAPK signaling pathway is shown in Figure 6.

The GSEA results showed that there were 13 KEGG pathways significantly enriched (*p* < 0.05, adjust *p* < 25%) in the ATR vs. C group, including 2 up-regulated gene sets and 11 down-regulated gene sets. Thereafter, eight of them were visualized, including up-regulated chaperones and folding catalysts (KEGGID:M03110) (Figure 5C) and seven down-regulated gene sets, namely, base excision repair (KEGGID:M03410), DNA replication (KEGGID:M03030), DNA replication proteins (KEGGID:M03032), nucleotide excision repair (KEGGID:M03420), cell cycle (KEGGID:M04110), translation factors (KEGGID:M03012) and DNA repair and recombination proteins (KEGGID:M03400) (Figure 5D). Ddetailed information is shown in Supplementary Table S3.

#### 3.4.5. Expression Profiles of Hsp Family Members

**Figure 7:**
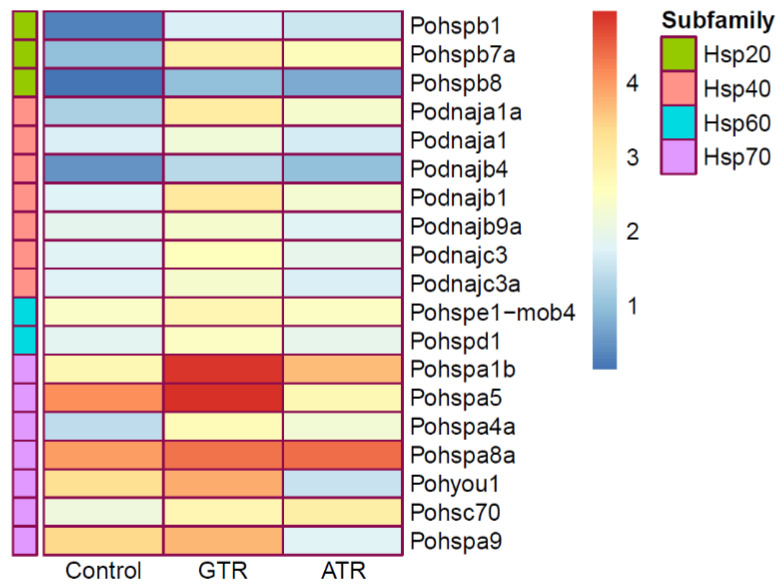
Expression profiles of *hsp (heat shock protein) 10/20/40/60/70* genes after heat stress. Each cell in the heat map corresponds to an expression level.

The expression levels of *hsp (heat shock protein) 10/20/40/60*/*70* family members were analyzed from the RNA-seq data and the heatmap is shown in Figure 7. Among *hsp10/20/40/60/70* family members, there were 19 DEGs which showed significant differences between the GTR group and the C group (*q*-value < 0.05 and |log2Foldchange| > 1), namely, 3 *hsp20* genes (*hspb1*, *hspb7a*, *hspb8*), 7 *hsp40* genes (*dnaja1*, *dnaja1a*, *dnajb1*, *dnajb4*, *dnajb9a*, *dnajc3*, *dnajc3a*), 2 *hsp10/60* genes (*hspe1-mob4*, *hspd1*) and 7 *hsp70* genes (*hspa1b*, *hspa5*, *hspa4a*, *hspa8a*, *hspa9*, *hsc70*, *hyou1*). All the DEGs were up-regulated significantly, and no down-regulated genes were found. After the abrupt temperature rise, nine DEGs were found, namely two *hsp20* genes (*hspb1*, *hspb7a*), three *hsp40* genes (*dnaja1a*, *dnajb1*, *dnajb4*) and four *hsp70* genes (*hspa1b*, *hspa4a*, *hspa8a*, *hsc70*). There were seven DEGs (*hspb1*, *dnaja1a*, *dnajb1*, *dnajb4*, *hspa1b*, *hspa4a*, *hspa8a*) found in both the ATR and GTR treatments.

## 4. Discussion

Antioxidant reactions could lead to histopathological alterations which reflect physiological and biochemical changes in individuals [75]. As critical respiratory and immune organs, as well as the primary sites of osmotic regulation and oxygen uptake, having direct contact with the aquatic environment, gills are more sensitive to environmental fluctuations, temperature especially [62,76]. Mallatt et al. first pointed out that higher temperatures often lead to gill alterations [77]. Since then, more and more studies have reported gill damage caused by heat stress. Heat shock could lead to lamellar fusion, lamellar aneurism and hyperplasia of gills in Japanese flounder [68]. In addition, heat stress was found to influence the gill histopathology of cichlid fish and pikeperch [78,79]. Except for heat stress, other stressors, including pesticides, could also cause morphological alterations in gill tissues [80]. In this study, we found that two kinds of heat shock led to gill damage in Japanese flounder, including lamellae swelling and obvious fusion of epithelial cells. In addition, we also examined the differences between the two stresses.

A steady-state ROS level is maintained by the production and elimination balance. However, the new generation of ROS induced by environmental factors, such as temperature, may destroy the balance. As a result, oxidative stress is stimulated and biomacromolecules, including DNA, are attacked by ROS [27,28,81]. Then, apoptosis is triggered when an individual cannot repair damaged DNA [82]. In this study, we measured the degree of DNA damage by evaluating the content of 8-O-hDG, and both GTR and ATR stress caused severe DNA damage. We hypothesize that the damage to DNA may be due to the increase in ROS levels. Similar results were also reported in other aquatic animals. DNA damage in pufferfish was shown to occur when the temperature rose to 34 °C [83], and increased apoptosis rates were confirmed in the hepatocytes of juvenile turbot after heat stress [83,84]. In addition, programmed cell death caused by heat stress was also reported in other aquatic animals, including American oyster and largemouth bass [84,85,86].

When experiencing oxidative stress caused by excessive ROS, individuals will activate the antioxidant system and enzymatic defense system to eliminate the toxic effects [87]. Antioxidant enzymes can maintain homeostasis and intracellular redox status in vertebrates [88,89,90,91]. Among them, SOD is considered to be vital in antioxidant processes [92]. In brief, SOD can catalyze the dismutation of superoxide radicals and prevent an organism from suffering further damage. SOD level is used to reflect antioxidant status, and the activation of SOD has been reported in the heat shock response of teleosts [79,84]. T-AOC level represents the whole antioxidant capabilities of an organism, including enzymatic antioxidants (e.g., SOD, CAT) and non-enzymatic antioxidants (e.g., GSH, ascorbate, hypotaurine) [93]. In previous studies, heat stress was found to damage the antioxidant system, and increased levels of biomacromolecule peroxidation were observed in Antarctic fish and turbot [84,94]. In the present study, the decrease in T-AOC levels in both the ATR and GTR stress groups were observed. We suggest that heat stress damages the antioxidant system in gill tissues and reduces antioxidant abilities in Japanese flounder. As a result, DNA damage as well as programmed cell death will be triggered subsequently.

Nowadays, next-generation deep sequencing (RNA-seq) has been frequently utilized in studies related to teleost evolution, development and stress reactions [95]. Numerous studies have illustrated the transcriptional responses of fish after heat stress, including sea bass, sea bream, large yellow croaker and zebrafish [96,97,98,99]. Transcriptomic techniques have also been used to examine the effects of temperature stress in Japanese flounder [65,66,67], but systematic studies are still lacking. In the present study, an RNA-seq analysis was utilized to elucidate the molecular mechanisms underlying the damages and changes in flounder physiology after heat stress. After screening DEGs, GO, KEGG and GSEA analyses were utilized to further explore gene functions; among these methods, the core of GSEA is to determine whether predefined sets of genes are differentially expressed in different phenotypes or different conditions [100]. Chaperone and folding catalyst pathways were the most significantly enriched pathways in KEGG and GSEA analyses. These were found to be increased after heat stress, indicating active refolding activities during reactions. In addition, this was closely connected to heat shock proteins, which was in accordance with the up-regulated *hsps* in DEGs, suggesting that the ratio of misfolded proteins decreased. Furthermore, DNA-related pathways (DNA replication-related terms, DNA repair-related terms, etc.) were also important. DNA damage sensing and repair proteins could be classified in the cellular stress response system [101]. Previous studies had illustrated that heat stress could influence DNA-related terms in zebrafish, half-smooth tongue sole and Japanese flounder [66,96,102]. In addition, 8-O-hDG is a good marker to estimate modulation of oxidative DNA damage and was found to be increased in teleosts after exposure to external stressors [103,104]. In this study, most DNA-related terms, especially DNA repair and replication-related terms, were decreased (*p* < 0.05), suggesting weakened repair efforts after heat stress. As a result, more DNA damage occurred and more 8-O-hDG was detected. Among the GO enrichment results, cell cycle is an important pathway that plays essential functions in the biological process. It has been found to be down-regulated after heat stress in this and other studies [105,106], hinting that heat stress affected the normal function of the cell cycle pathway of flatfish [66]. In addition, the apoptosis pathway was enriched and up-regulated in the ATR group, which might account for the TUNEL results. The functions of some gene sets need further in-depth study, such as cardiac muscle contraction.

As a ubiquitous and conserved signal transduction pathway, MAPK participates in numerous biological activities, including growth, development, and reactions to biotic and abiotic stressors from invertebrates to vertebrates [107,108,109,110,111]. In the previous studies carried out in our lab, three *mapk* genes were proved to participate in temperature response in the gill, heart, liver and spleen tissues of Japanese flounder [112]. *Mapk* genes were also demonstrated to react actively in other teleosts when exposed to temperature, salinity, hypoxia and bacterial challenges [113,114,115,116]. As shown in Figure 6, heat stress caused a cascade of responses: the elevation of ROS, the oxidative response and DNA damage. When the MAPK pathway was activated by DNA damage, there were three kinds of cell fate, and the hub genes showed different expression profiles in ATR and GTR. The first fate was apoptosis. In this case, JNK and Bcl XL, an important apoptosis factor, were down-regulated and up-regulated in GTR and ATR stress conditions, respectively, and both showed a more severe response in ATR stress. Our findings are in accordance with previous studies in other teleosts; *JNK* genes were proved to participate in salinity exposure in spotted gar [113]. In addition, JNK genes were up-regulated during perfluorooctane sulfonate exposure in the embryos of zebrafish, and this phenomenon had been linked to apoptosis in zebrafish larvae [117]. As the inhibition factor of Bcl, Bad had opposite expression profiles in GTR and ATR stress conditions. After ATR stress, the cell was more likely to experience this fate. The second cell fate was anti-apoptosis. The expression of anti-apoptosis factor IKK was up-regulated in GTR stress and down-regulated in ATR stress conditions, indicating that GTR stress activated the anti-apoptosis pathway while ATR stress inhibited the anti-apoptosis pathway. After GTR stress, the cell was more likely to experience the second fate. The last cell fate was proliferation and differentiation. In this case, MEK5 and ERK5 had opposite expression patterns in response to the two kinds of heat stress, while the expression of Nur77 was up-regulated in both GTR and ATR stress conditions. In other studies, significant up-regulation of JNK genes was reported and might be related to apoptosis in response to pollutant stressors [117]. After GTR stress, the cell was more likely to experience the third fate. As mentioned above, the MAPK signaling pathway showed diverse response patterns after GTR and ATR stress; ATR stress was more likely to cause the apoptosis cell fate. All the results above strengthened the important potential functions of *mapk* genes in the temperature stress reactions of Japanese flounder [66,67].

We used the expression profiles of *hsp10/20/40/60/70* family members of Japanese flounder as a representation of the enzymatic defense system in response to two kinds of heat stress utilizing the transcriptome. In unchallenged individuals, *hsps* are responsible for assisting with the folding of nascent proteins and preventing proteins from denaturing. When individuals are exposed to stressors, including high temperature, elevated expression of *hsps* could help organisms to resist stressors [118,119,120]. The results showed that 19 *hsp* genes responded significantly after GTR stress, while only 9 *hsp* genes responded after ATR stress. This indicated that chaperones were positively more affected by GTR stress than ATR stress. We hypothesize that the antioxidant system experienced slight damage and that repair was active after GTR stress, while the antioxidant system was destroyed seriously after ATR stress, resulting in a decrease in *hsp* gene activities. Heat stress often induces the expression of *hsp* genes; some *hsp40* genes, including *Hsp40A4* and *Hsp40B11*, were found to differentially expressed after heat shock in teleosts [43]. In this study, *hyou1* and *hspa5* of the *hsp70* subfamily were also observed to respond after GTR, which is in accord with previous findings [66]. Except for Japanese flounder, *hsp* superfamily members were found to respond actively to heat stress in other aquatic animals [37,39,40,42,121]. After one-hour heat stress at 37 °C, the *hspb1* gene of zebrafish was found to be up-regulated significantly; similar differential expression patterns of *hspb1* were also reported in mammalian systems, which hinted at the potential responsibility of the *hspb1* gene in protecting organisms from being damaged by heat shock [42,122]. As mentioned above, the differential expression of *hsp10/20/40/60/70* family members suggests that *hsp* genes were induced actively to protect Japanese flounder from being damaged. Further studies are required to examine in more detail the molecular mechanisms involved in heat shock response in Japanese flounder in order to protect this economically important marine species from damage due to heat stress and to breed heat-tolerant strains.

## 5. Conclusions

In this study, adult Japanese flounder were subjected to two kinds of heat stress (GTR and ATR stress). Different degrees of damage were observed and more severe injury was found after ATR stress. Temperature rises caused oxidative stress, increased ROS levels and 8-O-hDG levels and reduced T-SOD activities and T-AOC levels, hinting at the destruction of the antioxidant system which led to programmed cell death. Transcriptome results showed that Japanese flounder respond to oxidative stress by enhancing the activity of chaperone and folding catalyst pathways, the MAPK signaling pathway and *hsp10/20/40/60* genes. In short, more severe damage and weakened repair efforts were observed after ATR stress than GTR stress. Our findings provide useful information about the molecular mechanisms underlying heat shock reactions in Japanese flounder and deepen understanding of the transcriptomic and physiological responses to heat stress in fish.

## Figures and Tables

**Table 1:** The DEG (differentially expressed gene) numbers after two kinds of heat stress.

Term	GTR vs. C	ATR vs. C
Up-regulated	341	627
Down-regulated	166	423
Total	507	1060

**Table 2:** GO (gene ontology) enrichment results after the gradual temperature rise (GTR).

GO ID	*p*-Value	Count	Term	Description	Classification
0070417	0.0154	1	Cellular response to cold	Up-regualted	Biological Process
1990440	0.0154	1	Positive regulation of transcription from RNA p Polymerase II promoter in response to endoplasmic reticulum stress	Up-regulated	Biological Process
0043620	0.0139	2	Regulation of DNA-templated transcription in response to stress	Up-regulated	Biological Process
0002526	0.0163	2	Acute inflammatory response	Up-regulated	Biological Process
0002190	0.0187	1	Cap-independent translational initiation	Down-regulated	Biological Process
0006260	0.0000	5	DNA replication	Down-regulated	Biological Process
0006281	0.0098	3	DNA repair	Down-regulated	Biological Process
0045004	0.0094	1	DNA replication proofreading	Down-regulated	Biological Process
0006268	0.0094	1	DNA unwinding involved in DNA replication	Down-regulated	Biological Process

**Table 3:** GO (Gene Ontology) enrichment results after the abrupt temperature rise (ATR).

GO ID	*p*-Value	Count	Term	Description	Classification
0002526	0.0163	2	Acute inflammatory response	Up-regulated	Biological Process
0036269	0.0268	1	Swimming behavior	Up-regulated	Biological Process
0005149	0.0497	1	Interleukin-1 receptor binding	Up-regulated	Molecular Function
0033549	0.0045	2	MAP kinase phosphatase activity	Down-regulated	Molecular Function
0036310	0.0394	1	Annealing helicase activity	Down-regulated	Molecular Function
0071140	0.0268	1	Resolution of mitotic recombination intermediates	Down-regulated	Biological Process
0006259	0.0000	17	DNA metabolic process	Down-regulated	Biological Process
0006261	0.0000	10	DNA-dependent DNA replication	Down-regulated	Biological Process
1990423	0.0301	1	RZZ complex	Down-regulated	Cellular Component
0030894	0.0000	4	Replisome	Down-regulated	Cellular Component
0005657	0.0000	6	Replication fork	Down-regulated	Cellular Component
0033186	0.0301	1	CAF-1 complex	Down-regulated	Cellular Component
0031390	0.0301	1	Ctf18 RFC-like complex	Down-regulated	Cellular Component

**Table 4:** KEGG enrichment results after the gradual temperature rise (GTR).

KEGG ID	*p*-Value	Count	Term	Description
3110	3 × 10^−28^	40	Chaperones and folding catalysts	Up-regualted
3018	2 × 10^−3^	7	RNA degradation	Up-regulated
4091	1 × 10^−2^	5	Lectins	Up-regulated
3032	2 × 10^−16^	21	DNA replication proteins	Down-regulated
3410	1 × 10^−9^	9	Base excision repair	Down-regulated
4110	1 × 10^−4^	10	Cell cycle	Down-regulated
3420	8 × 10^−4^	5	Nucleotide excision repair	Down-regulated
3430	3 × 10^−2^	6	Mismatch repair	Down-regulated
3460	5 × 10^−2^	3	Fanconi anemia pathway	Down-regulated

**Table 5:** KEGG enrichment results after the abrupt temperature rise (ATR).

KEGG ID	*p*-Value	Count	Term	Description
3110	2 × 10^−9^	22	Chaperones and folding catalysts	Up-regualted
4210	4 × 10^−2^	8	Apoptosis	Up-regulated
3018	5 × 10^−2^	5	RNA degradation	Up-regulated
3032	2 × 10^−18^	23	DNA replication proteins	Down-regulated
3410	1 × 10^−9^	9	Base excision repair	Down-regulated
4110	8 × 10^−7^	13	Cell cycle	Down-regulated
3036	7 × 10^−4^	32	Chromosome and associated proteins	Down-regulated
3420	9 × 10^−4^	5	Nucleotide excision repair	Down-regulated
4050	7 × 10^−3^	5	Cytokine receptors	Down-regulated
3430	3 × 10^−2^	2	Mismatch repair	Down-regulated

## Data Availability

Publicly available datasets were analyzed in this study. This data can be found in NCBI in the accession number: PRJNA716811, PRJNA717095 and PRJNA776544.

## Supplementary Materials

The following supporting information can be downloaded at: https://www.mdpi.com/article/10.3390/ijms23063286/s1.
