# Supplementary material for: Molecular Mechanism Based on Histopathology, Antioxidant System and Transcriptomic Profiles in Heat Stress Response in the Gills of Japanese Flounder
Source: Int J Mol Sci. 2022 Mar 18;23(6):3286. doi: 10.3390/ijms23063286 (PMC8955770; doi:10.3390/ijms23063286)
Supplement: Supplementary file 1 [file ijms-23-03286-s001.zip › ijms-1629416-supplementary.pdf]

Table S1 The statistic of gill transcriptome after heat stress

Group	Type	Count	Group	Type	Count	Group	Type	Count
GTR-1	Raw reads	59602370	ATR-1	Raw reads	45364792	C-1	Raw reads	44436390
	Clean reads	58468750		Clean reads	44018696		Clean reads	43667310
	Clean reads rate	98.1%		Clean reads rate	97.03%		Clean reads rate	98.27%
	clean bases	8.77G		clean bases	6.6G		clean bases	6.55G
	Q20	97.69%		Q20	97.37%		Q20	97.76%
	Q30	93.68%		Q30	92.99%		Q30	93.76%
	GC	47.74%		GC	48.99%		GC	48.7%
	Total mapped reads to genome	90.89%		Total mapped reads to genome	87.19%		Total mapped reads to genome	84.74%
GTR-2	Raw reads	46419412	ATR-2	Raw reads	42680686	C-2	Raw reads	66225858
	Clean reads	45593988		Clean reads	41564046		Clean reads	65312560
	Clean reads rate	98.22%		Clean reads rate	97.38%		Clean reads rate	98.62%
	clean bases	6.84G		clean bases	6.23G		clean bases	9.8G
	Q20	97.76%		Q20	97.65%		Q20	97.65%
	Q30	93.79%		Q30	93.49%		Q30	93.56%
	GC	47.55%		GC	48.61%		GC	47.77%
	Total mapped reads to genome	87.63%		Total mapped reads to genome	91.02%		Total mapped reads to genome	91.12%
GTR-3	Raw reads	47225966	ATR-3	Raw reads	49153778	C-3	Raw reads	49037112
	Clean reads	46502188		Clean reads	47585362		Clean reads	47759732
	Clean reads rate	98.47%		Clean reads rate	96.81%		Clean reads rate	97.4%

clean bases	6.98G	clean bases	7.14G	clean bases	7.16G
Q20	97.89%	Q20	97.57%	Q20	97.69%
Q30	94.08%	Q30	93.43%	Q30	93.68%
GC	48.64%	GC	48.93%	GC	47.67%
Total mapped reads to genome	88.23%	Total mapped reads to genome	85.95%	Total mapped reads to genome	89.39%

Table S2 The GSEA annotation results after Gradual Temperature Rise (GTR)

Name	KEGG ID	p value	p. adjust	Description
Chaperones and folding catalysts	M03110	0.001328021	0.082437276	Up-regulated
G protein-coupled receptors	M04030	0.008526188	0.130734876	Up-regulated
MAPK signaling pathway	M04040	0.001236094	0.082437276	Up-regulated
Protein processing in endoplasmic reticulum	M04141	0.001344086	0.082437276	Up-regulated
Cytoskeleton proteins	M04812	0.002306805	0.102678571	Up-regulated
Cardiac muscle contraction	M04260	0.005738881	0.105595409	Up-regulated
Neuroactive ligand-receptor interaction	M04080	0.014652015	0.174242424	Up-regulated
DNA replication proteins	M03032	0.003378378	0.102678571	Down-regulated
Spliceosome	M03040	0.003436426	0.102678571	Down-regulated
Ribosome biogenesis	M03009	0.00390625	0.102678571	Down-regulated
Steroid biosynthesis	M00100	0.004950495	0.105383734	Down-regulated
DNA replication	M03030	0.005154639	0.105383734	Down-regulated
Base excision repair	M03410	0.0078125	0.130681818	Down-regulated
Ribosome biogenesis in eukaryotes	M03008	0.012195122	0.17260788	Down-regulated
DNA repair and recombination proteins	M03400	0.013888889	0.174242424	Down-regulated
Peptidases and inhibitors	M01002	0.015151515	0.174242424	Down-regulated

Table S3 The GSEA annotation results after Abrupt Temperature Rise (ATR)

Name	KEGG ID	p value	p.adjust	Description
Chaperones and folding catalysts	M03110	0.002528445	0.106321839	Up-regulated
Neuroactive ligand-receptor interaction	M04080	0.017523364	0.237382378	Up-regulated
Base excision repair	M03410	0.002688172	0.106321839	Down-regulated
DNA replication	M03030	0.002777778	0.106321839	Down-regulated
Glycosylphosphatidylinositol (GPI)-anchored proteins	M00537	0.003003003	0.106321839	Down-regulated
DNA replication proteins	M03032	0.003448276	0.106321839	Down-regulated
RNA transport	M03013	0.004385965	0.115914787	Down-regulated
Nucleotide excision repair	M03420	0.005681818	0.131392045	Down-regulated
Cell cycle	M04110	0.007874016	0.161854768	Down-regulated
C-type lectin receptor signaling pathway	M04625	0.010909091	0.201818182	Down-regulated
Translation factors	M03012	0.013793103	0.230099502	Down-regulated
Cytokine receptors	M04050	0.014925373	0.230099502	Down-regulated
DNA repair and recombination proteins	M03400	0.017964072	0.237382378	Down-regulated
